# Diagnosis and Management of Silicotuberculosis in Resource-Limited East Africa

**DOI:** 10.7759/cureus.76961

**Published:** 2025-01-05

**Authors:** Jacob T Harris, Kaleb Ward, Alexandra Winski, Emmanuel Elisha, Mlungwana G Lukwaro, Zablon Muterian, Kei Nagatomo, Catherine Wagner, Priya Radhakrishnan

**Affiliations:** 1 Internal Medicine, HonorHealth, Scottsdale, USA; 2 Family Medicine, HonorHealth, Scottsdale, USA; 3 Internal Medicine, Oltrumet Hospital, Arusha, TZA; 4 General Surgery, HonorHealth, Scottsdale, USA; 5 Academic Affairs, HonorHealth, Scottsdale, USA; 6 Internal Medicine and Academic Affairs, HonorHealth, Scottsdale, USA

**Keywords:** global health, public health, silicotuberculosis, silicotuberculosis and silicosis, silicotuberculosis and tuberculosis, silicotuberculosis diagnosis, silicotuberculosis in east africa, silicotuberculosis in resource-limited areas, silicotuberculosis treatment

## Abstract

A 41-year-old East African man with a history of previously treated tuberculosis (TB) presented with hemoptysis, dyspnea, night sweats, and fatigue. He was treated for assumed silicotuberculosis (ST) and made a good recovery. The diagnosis of ST is challenging and requires thorough evaluation due to its overlapping clinical and radiological features with pulmonary TB and silicosis. Its diagnosis and treatment in resource-limited areas is especially complicated and nuanced. Understanding the effects of resource limitation on the diagnosis and treatment of ST can help the medical community address the medical care gap often present in these areas of the world since the region's limited access to advanced diagnostic tools and treatment options exacerbates the burden of the disease.

## Introduction

Silicotuberculosis (ST) is a rare condition worldwide that is characterized by the coexistence of silicosis and tuberculosis (TB). Its overall prevalence is difficult to determine due to the extreme variability based on the demographics studied. Silicosis alone is more common, especially among mining communities, and its prevalence depends on the location and type of mining. In general, the prevalence of silicosis among mining communities ranges from 18% to as high as 28% [1-3]. In comparison, the prevalence of ST is estimated at 3-7% in mining communities and 0.3-2.3% in the general population in East Africa [4].

Silica exposure increases the risk of TB, with the relative risk of developing pulmonary TB in patients with silicosis compared to patients without silicosis being estimated at 2.8 [5]. This means that the presence of silicosis, in addition to TB, makes a patient almost three times more likely to develop pulmonary TB when compared to TB patients without silicosis. Specifically, silica exposure impairs inflammatory response through diminishing dendritic cell activation [6], downregulates Toll-like receptor 2 [7], and increases intracellular replication and macrophage release of TB bacteria [8]. The diagnostic criteria for ST include the clinical/radiographic presence of silicosis and the microbiologic presence of TB. Elevated immunological markers also support the diagnosis [9]. Treatment includes the standard of care for TB, which is sometimes extended to eight months due to the increased risk of relapse in the cases of ST compared to typical TB. Prophylactic isoniazid is also recommended for this reason [10]. In resource-limited environments, the diagnosis and treatment of ST is more nuanced and relies more heavily on clinical assessment. This paper will discuss the diagnosis and treatment of ST in the setting of resource-limited countries.

## Case presentation

A 41-year-old East African man presented to the hospital in his country of origin for hemoptysis, dyspnea, night sweats, and fatigue lasting for two months. Medical history includes TB diagnosed four months prior with TB microscopy and treated with rifampin, isoniazid, pyrazinamide, and ethambutol. Previously, he had more severe symptoms of hemoptysis, dyspnea, night sweats, and fatigue. He took his medications as prescribed. Additional information regarding his medical history was difficult to acquire due to a lack of continuity in the medical record. At the time of hospitalization, he was taking dual therapy of rifampin and isoniazid. The patient also worked for seven years in a local gemstone mine for employment. In the area/mines where he worked, exposure to silica was prevalent during the process of gem mining.

The initial vital signs showed an oxygen saturation of 75%, a temperature of 38.9 °C, and a blood pressure of 136/68. A physical exam showed an ill-appearing man with coarse crackles and wheezing in bilateral lung fields with labored breathing and poor air movement. The patient was euvolemic, and the cardiovascular exam was unremarkable, with no jugular venous pressure elevation. The abdominal exam and skin exam were also normal. The initial TB testing performed using the Xpert *Mycobacterium tuberculosis*/rifampin diagnostic test (GeneXpert MTB/RIF, Cepheid Inc., Sunnyvale, California) was positive, which was expected given his prior TB infection. TB microscopy testing for acid-fast bacilli was subsequently performed but was negative. The patient was, therefore, continued on his TB treatment of rifampin and isoniazid. Prednisolone was added to treat possible interstitial lung disease. A community-acquired pneumonia diagnosis was entertained, but antibiotics for such were later discontinued. The patient was ultimately diagnosed with assumed ST, given his history of working in the gemstone mine with silica exposure (with an initial TB diagnosis prior to admission). A chest X-ray was unable to be obtained due to resource scarcity. Additional symptomatic treatment was provided, and the patient gradually improved. His symptoms subsided, and his oxygen saturation improved to near-normal levels.

## Discussion

The case presented here represents a likely case of ST and highlights the difficulties in diagnosing and treating ST in resource-limited environments. ST is diagnosed through a combination of clinical assessment, radiographic imaging, and microbiologic blood work. Chest imaging with X-rays or high-resolution CT is often used to visualize silicosis and TB lesions, which are frequently described as nodular opacities, usually in the upper lung lobes. The diagnosis of TB is further obtained through sputum microscopy, culture, and nucleic acid amplification tests [9].

Of note, in patients with known silicosis cases, the T-SPOT test is more effective than the tuberculin skin test in diagnosing TB [11]. The various TB diagnostic tests and their respective sensitivities and specificities are included in Table 1. Elevated immunological markers such as immunoglobulin E (IgE), immunoglobulin G (IgG), fibronectin, CD4 T lymphocyte surface protein, and B-lymphocyte antigen CD20 further support the diagnosis of ST [9]. The World Health Organization's recommendations for diagnosing TB include an algorithmic approach progressing from a four-symptom screening to molecular rapid testing (e.g., GeneXpert MTB/RIF) and bacteriological confirmation [12]. Local African guidelines make similar recommendations of sputum smears/molecular tests (e.g., GeneXpert), microbiological culture, and chest X-rays [13]. Screening for TB is recommended for all patients with silicosis since these patients have a fourfold [14] to 39-fold [10] increase in TB risk compared to patients without silicosis.

**Table 1:** Tuberculosis diagnostic test sensitivities and specificities Xpert MTB/RIF: Xpert *Mycobacterium tuberculosis*/rifampin diagnostic test; Xpert MTB/RIF Ultra: Xpert *Mycobacterium tuberculosis*/rifampicin ultra; Real-Time PCR: Abbott RealTime Mycobacterium Tuberculosis Assay

Diagnostic Test	Sensitivity	Specificity
Tuberculin Skin Test [19]	86.5% (at 5 mm cutoff)	61.6% (at 5 mm cutoff)
	81.7% (at 10 mm cutoff)	71.3% (at 10 mm cutoff)
	55.6% (at 15 mm cutoff)	88.0% (at 15 mm cutoff)
QuantiFERON-TB Gold-in-Tube [19]	77.7% (at 0.35 IU/mL)	98.3% (at 0.35 IU/mL)
	66.9% (at 0.7 IU/mL)	99.1% (at 0.7 IU/mL)
	61.5% (at 1.0 IU/mL)	99.4% (at 1.0 IU/mL)
T-SPOT [19]	79.2% (at 5 spots)	96.7% (at 5 spots)
	76.7% (at 6 spots)	97.7% (at 6 spots)
	74.0% (at 7 spots)	98.6% (at 7 spots)
	71.8% (at 8 spots)	99.5% (at 8 spots)
Xpert MTB/RIF [20]	89%	98%
Xpert MTB/RIF Ultra [21]	88.90%	95.60%
Mycobacterial Culture [22]	60%	~100%
Smear Microscopy [22]	22%	~100%
Chest Radiography [22]	64%	78%
Loop-Mediated Isothermal Amplification [20]	93%	94%
Simultaneous Amplification Testing [20]	96%	88%
Real-Time PCR [23]	85.70%	100%

The treatment of ST is typically the same as first-line anti-TB quadruple therapy, as seen in the patient above. This includes rifampin, isoniazid, pyrazinamide, and ethambutol for two months, followed by rifampin and isoniazid for four months (although dual therapy can be extended to 10 months for extrapulmonary TB) [13]. Typical treatment for TB alone lasts at least six months. However, due to the increased risk of disease relapse and possible drug reactions in cases of concurrent silicosis, treatment time in patients with ST is extended to at least eight months [6]. Evidence that patients with ST have greater than two times the odds of poor treatment outcomes compared to patients without silicosis also supports this extended time course of treatment [15]. This is also similar to the treatment course for patients with immunocompromised conditions. The long-term use of isoniazid for chemoprophylaxis can be utilized to minimize these same risks. Second-line therapies for TB can be used in cases of drug resistance. Numerous types of drug resistance exist in TB, for which various therapies are available; however, this discussion is beyond the scope of the current study.

The diagnosis and treatment of ST are more nuanced in resource-limited areas. The reason for this is multifactorial but includes limited access to or delay in standard diagnostic tools, inconsistent treatment adherence due to socioeconomic factors, comorbid conditions that may be uncontrolled, limited infrastructure to provide comprehensive care, and medical education that is not updated with the latest advancements and research [16]. Inconsistent access to electricity amplifies these difficulties. While treatment does center around anti-TB regimens, ensuring adequate care is made difficult by job insecurity, food insecurity, and high patient geographic mobility [17]. While employment status provided a socioeconomic advantage in the case of our patient, diagnostic tools, comprehensive care, and electricity limitations were specifically relevant in his care.

Nevertheless, despite the limitations outlined above, diagnosis and treatment of ST can still be achieved in resource-limited areas. For example, although it has relatively limited sensitivity, sputum smear microscopy has been demonstrated to be a cost-effective and straightforward diagnostic alternative. Urinary lipoarabinomannan detection of mycobacterium in the urine can be effective, especially in patients with human immunodeficiency virus (HIV) and low CD4 counts. A fine-needle aspiration biopsy is an alternative that can be particularly helpful in diagnosing extrapulmonary TB. Finally, interferon-gamma release assays can also be utilized [18].

Treatment in resource-limited countries can also be affected by finite supplies in hospitals and homes. Oxygen supplementation is a common element of treatment, but extra canisters can be difficult to deliver to the hospital during times of high demand. Home oxygen is challenging to obtain for similar reasons. Sometimes, the disconnect between patients' incomes and the cost of medicines makes medication adherence a lower priority, and inadequate treatment increases. Consistent clinical monitoring during and after treatment can be difficult when patients live large distances from the hospital and can only make the journey on foot. For these reasons and many others, the challenges of treatment remain significant barriers to optimal treatment in resource-limited areas.

## Conclusions

ST is a rare condition that is more prevalent in areas of mining communities and endemic TB. Its diagnosis and treatment can be relatively standardized but are complicated in the settings of resource limitation and social determinants of health. In these areas, alternatives in diagnostic methods can be utilized, but treatment remains centered around anti-TB therapies. This complication is partly due to delays in standard diagnostic tools, inconsistent treatment adherence, uncontrolled comorbid conditions, limited hospital/clinic infrastructure, and divergent medical education. An increased understanding of the consequence of resource limitation on the diagnosis and treatment of ST can help the medical community address the medical care gap often present in these areas of the world.
